# Physiological and Pathological Observations on the Sympathetic Disorders and Consecutive Diseases of Dyspepsia

**Published:** 1831-12

**Authors:** William John Thomas


### DYSPEPSIA.

### *Physiological and Pathological Observations on the Sympathetic Disorders and Consecutive Diseases of Dyspepsia.*

By WILLIAM JOHN THOMAS, M.R.C.S.

THE innumerable and ever-varying symptoms attendant upon derangement of digestion, defy all attempts at systematic classification. Some excellent papers have been written upon the subject, and much information has been laid before the medical public; and yet we are compelled to confess that a considerable hiatus yet exists even in the historical delineations of the disease; many peculiarities remain unnoticed, and much further information has yet to be supplied before we can obtain a correct view of the nature of the disease in question.

After the excellent work of Dr. JOHNSON, which I consider the best extant, we may indeed be pardoned in supposing that little further might be required for the elucidation of the subject. That this gentleman has written well, is universally admitted by all competent judges, and the high satisfaction which I have derived from the perusal of his practical work demands a public recognition of its excellencies, when treating upon a similar subject. My attention has been, for some years back, directed to the subject of derangement of digestion, in consequence of much painful experience of its distressing peculiarities. Having had the unenviable opportunity of scrutinizing the phenomena of indigestion in my own person, I endeavoured to trace in my patients the progress of similar symptoms, and sympathies of an identical nature; in consequence of which investigation, I

have deemed it expedient to throw out a few remarks upon the subject, trusting that they may be acceptable to your readers. It is not my intention to make many minute observations upon the most remarkable symptoms; for these, being subjects of daily observation, may not demand so precise a consideration. The eructations of biliary matter, the formation of acid, and the distention of the stomach by containing gases, may, however, require a few observations.

The remarkable phenomena of the formation of acid in the stomach may be adverted to, in the first place. When a dyspeptic invalid has partaken of his ordinary food, the formation of acid commences: I have remarked, in several instances, that scarcely ten minutes have elapsed from the reception of the food before the acid is perceptible. Some physiologists have stated that the acid is the muriatic, and that it is secreted by the vasa vasorum of the stomach, and the arterial capillaries which these minute vessels supply; but I am convinced that in many instances the nature of the acid is materially modified, and that occasionally, instead of an acid, an alkali is produced.

The great question which has hitherto eluded our inquiries is this, by what peculiar process is this acid produced? Is it secreted, or can it be the produce of fermentation? From a number of observations, and careful investigations of the subject, I have come to the conclusion that this acid is derivable from three distinct sources: first, I am convinced that a fermentation actually takes place in the *diseased* stomach; secondly, I believe that the arterial ramifications occasionally secrete an acid fluid; and, lastly, I apprehend that the production of either acid or alkali may be referred to a chemical decomposition of the ingesta: the primary elements of the food may combine together, and by their combinations form the new constituents of alkaline or acid principles. In the majority of dyspeptic cases, the appetite is good, a craving for food frequently takes place, and faintness will ensue if the demands of nature be not promptly complied with. The result of this morbid appetite is, that the patient partakes too freely of food, and, forgetting the diseased condition of the organ, he distends the stomach until the craving void in the epigastrium is filled up to satiety. The stomach thus becomes morbidly distended with solid ingestum, which immediately paralyses the motions of that viscus, and the phenomena of indigestion appear. The stomach being distended with flatus, the cardiac sphincters are violently burst open, and the eructations of the generated gases ensue. It would be a most desirable experiment if any of our minute chemical philosophers, who have so often illuminated the scientific world by their practical elucidations of complex chemical phenomena, would undertake to analyse these gases. The airs to be analysed might be conveniently received over mercury, and their primary elements and combinations accurately ascertained. A patient who has long laboured under derangement of digestion could readily, at any time after a repast, furnish sufficient materials of an aëriform nature for the analysis of the scientific investigator. The physiologist might thus ascertain whether the gases produced bore any relative proportion to the primary elements of the food: he might compute the different proportions of the different gases produced by different species of food, and, by this proceeding, in some measure decide the important question whether these invisible agents are actually produced from the food, or secreted from the capillary apertures of the arterial exhalents. From the experiments on digestion instituted by Dr. Wilson Philip and others, we might conclude that the electricity of the brain or nervous fluid exercises a considerable influence over the functions of digestion: but experiments of this description, however laudable the motive for performing them may be, must necessarily be subject to many objections: first, we may consider that an accurate analogy can never be drawn from these experiments upon brutes, and applied to human beings, because we find that a great distinction exists between the two classes of beings. We all know that beasts and reptiles of the earth select food for themselves, which to them is perfectly salutary, but which to man would be pernicious, deleterious, and poisonous. Secondly, we cannot with propriety suppose that we are investigating the *natural* action of the stomach, when we first cut through important parts to arrive at vital organs, with which the stomach must of necessity sympathise, and which sympathy must originate a disordered process in equal ratio of activity to the sympathetic intensity. The learned and accomplished physiologists who make these experiments may, indeed, prevent vomiting by tying the œsophagus, but I do not perceive how such violent proceedings can possibly procure for us an opportunity of investigating the *natural* process of digestion. The distention of the stomach having proceeded to an extent which cannot be increased, the diaphragm and abdominal muscles contract; the stomach becomes affected with spasm; the pylorus is pushed inwards towards the duodenum, and its orifice is torn open: the gaseous and fluid materials are thus protruded into the duodenum. When this bowel is preternaturally distended with air or increment, the ductus choledochus communis is, of necessity, acted upon by the physical agents, and, from the irritation produced by the distention, the gall-bladder becomes affected with spasm, and bile is thrown out profusely into the duodenum. A specific irritation having been already introduced into this viscus by the acrimonious chyme from the stomach, the pylorus is preternaturally affected, and no longer performs the office of a valve; the contents of the duodenum are therefore regurgitated into the stomach, and this unfortunate organ has now the advantage of the extraordinary stimulus of the bile, added to its own original acrimonious contents.

At this stage of the disorder, some remarkable phenomena generally arise. In the first place, a sympathetic pain is perceived in the occiput: this pain is described as that of a dull, obtuse, and vibratory sensation. In many cases, the affections of the head and stomach alternate in reciprocal participation of morbid action. I shall illustrate this position by a remarkable case: A gentleman complained of this dyspeptic cephalagia; it commonly occurred after dinner, when the biliary affections had previously prevailed; a great degree of acidity was produced in the stomach after each repast, and he was accustomed to neutralise the acid with large doses of the bicarbonate of soda. Upon the neutralization of the acid, the gastric irritation subsided; but the morbid action was transferred to the cerebellum, as the occipital sensations appeared to indicate. In this case the following experiment was instituted: The patient was instructed to neutralise the acid as usual with the bicarbonate, and, if the irritation of the brain succeeded, to plunge the head into a pailful of cold spring water. As usual after dinner, the acid was formed in the stomach, and when the eructations of concentrated acid, bile, and flatus proclaimed the prevalence of gastric irritation, he swallowed several doses of the bicarbonate: in the space of five minutes, the disorder of the stomach subsided, and the headach ensued. After the latter symptom had continued for about half an hour, the patient plunged his head into cold water, and experienced an instantaneous relief from the headach. Whilst he was congratulating himself upon the efficacy of this new remedy, the stomach became affected a second time, and the contents vomited partook of the acerbity of "oil of vitriol:" the bicarbonate was again employed, and again the headach supervened. The cephalagiac paroxysm became so intense, that the patient had a second time recourse to the cold bath, to alleviate or repel the pain, when precisely the same symptoms of gastric acidity reappeared, and the unhappy patient threw himself upon the couch in the anguish of unspeakable despair.

During these severe paroxysms of gastric irritability, a cloud of melancholy suspends its deep shadows over the agitated mind; a dejection of thought, with dark delineations of the prospects of futurity, takes place, and melancholy extends the black mantle of despair over the ideal felicities which had formerly flourished in the sunshine of mental serenity. The patient, under these impressions, invariably views his finances in the least advantageous light, and, although his coffers may be filled with gold, yet he will imagine himself upon the point of pecuniary insolvency. It will readily be admitted that these accumulated irritations in the stomach cannot long exist without the adjacent parts partaking of the disorder; and accordingly we find a tenderness upon pressing the epigastrium, extending over the hypochondriac regions. This pain upon pressure is not exactly indicative of inflammation; in many instances it appears the result of proximate irritation alone, for the pain subsides as the dyspeptic paroxysms disappear. This spontaneous subsidence of the fulness and tenderness of the epigastrium is, in my opinion, diagnostic of the earlier stages of dyspepsia: so long as the pain upon pressure exists merely in the form of paroxysm, we need not fear any actual disorganization of the coats of the stomach. I have remarked that, when the dyspeptic irritation attacks the bowels, which is frequently the case, the irritation in the stomach subsides; but if the purging be incautiously arrested, by the use of opiates, &c., the original affection of the gastric organ reappears. The same may be remarked of the strangury which occasionally appears as a dyspeptic symptom: it intermits with the stomachic irritation, and the immediate developement of the one is the signal for the extinction of the other.

This fact peculiarly illustrates the axioms of physiology; for, whenever the heart, the liver, the brain, or the bowels, are sympathetically affected in this complaint, the disorder is confined to *one* viscus only; and we find that two distinct organs are never sympathetically affected at the same moment, unless the irritation has proceeded to an extraordinary degree of intensity, and whenever several viscera are symptomatically assailed by permanent irritation at the same time, we may rest assured that organic disease exists in the viscus primarily irritated, and also not unfrequently in the organs participating in a preternatural action, which has become permanently sympathetical.

That these irritations terminate ultimately by inflammation is a maxim which daily experience compels us to admit; for, physiologically speaking, it is scarcely possible that the minute ramifications of nerves should be preternaturally excited for an indefinite time, without losing a portion of their peculiar powers or energies; and if they thus, by preternaturally excited action, lose a portion of their natural powers, the arterial ramifications, over whose specific action they continually preside, must necessarily be affected by the loss of nervous power. Thus, I apprehend, it will be generally admitted that the minute branches of nerves expanded over the serous tissues, and other delicate membranes, preside, in an especial manner, over the arterial and exhalant ramifications of those peculiar textures; and that the contractility and expansibility of the capillary organs are in equal ratio to the momentum of nervous impression. Let us then suppose, that, by preternatural excitement, the stimulating powers of these delicate nerves are at a discount, the necessary result will be, that the contractility of the arterial trains will become less intense; and the inferential corollary, that the balance between the exhalent and absorbent systems will be ultimately destroyed. When we consider that the electricity of the nerves is the principal cause of the diversity of the chemical phenomena of the secretions, we may expect that the materials exhaled from the arteries will be vitiated in their natural composition, and originate that permanent proximate reaction which ends only in the disorganization of the textures acted upon.

It is not my intention, in this rapid sketch of the consecutive symptoms of dyspeptic irritations and sympathies, to enter into the why and wherefore of the prerogative of nerves in the composition and decomposition of textures, but I would briefly remark, that, since all the materials of the body are deducible from the blood, the great modification of the constitutions of those textures compels us to admit that some formative agent must necessarily operate upon the unity of the primary materials; and we may infer that a galvanic modification of nervous power is sufficient to account for the phenomena of organization. I would not, however, be understood to maintain that this galvanic principle is the sole agent in the crystallization of the osseous, fibrous, or cartilaginous laminæ or fasciculi; but that I am firmly convinced that new complications of primary elements ensue at the extremity of the capillary exhalents, and these modifications of the secreted fluids, and the new arrangements of the primary elements, are caused, in a great degree, by the immediate operation of nervo-galvanic power upon the acuminated extremities of the exhalents. In this manner we may account for the ossification of the valves of the heart, which frequently occurs when the dyspeptic sympathies have for some time exercised themselves over that important organ. That spasms of the heart frequently arise from derangement of digestion, is a statement which will never be questioned by those who have had opportunities of remarking the progress of dyspeptic sympathies: the spasm invariably takes place towards evening, and the patient is frequently aroused out of his slumbers by an alarming apprehensibility of suffocation caused by the tumultuous action of the heart. A slow and obscure action of the exhalents is, however, a symptom of a more dangerous nature, and, by the derangement of the nervous power, these vessels secrete the bony spiculæ which cover the valves of the heart. I well remember a case wherein I severely wounded my fingers, by incautiously introducing them through the auriculo-ventricular opening during a post-mortem inspection; the valves being consolidated by bony laminæ and osseous projections, of a needle-like appearance. I may briefly advert to phthisis arising from the gastric irritation, as an example of the effect of this great morbid cause in originating consumption of a most fatal character. This disease may or may not be accompanied with expectoration, according to the intensity of the primary and consecutive irritation; but, whether the discharge be profuse or otherwise, the disease is always attended with marasmus and hectic fever. In some stages of dyspeptic phthisis, I have remarked that the tongue is coated with a semipellucid pellicle, of a greyish substance, resembling coagulable lymph. The majority of cases of consumption of the lungs originating from dyspeptic irritation, terminate either by diarrhœa or typhoid fever.

It will be readily acknowledged, from the preceding observations, how exceedingly necessary it is to treat in a prompt and scientific manner the biliary disorders which originate these fatal maladies; and this observation is the more necessary, since, although the primary irritation may be allayed by the use of medicine, yet, upon the slightest imprudence in diet, the disorder will be instantly aroused. The volumes that have been written upon diseases of the liver, the spleen, the kidneys, and circumjacent viscera, attest, in a peculiar degree, the necessity of attention to the action of that fountain of all nutrition, the stomach; for we cannot expect the streams to be pure when the sources which supply them are contaminated.

I should exceed the bounds of a few observations were I

to enumerate the multitude of symptoms, morbid sensations, and nervous shocks that alarm the susceptible minds of dyspeptic invalids: sufficient has, however, been said to keep alive the attention of the medical world to one of the most powerful exciting causes of dormant constitutional affections, to the primary cause of the developement of a number of local diseases, and to a subject whose peculiar interest consists in the incontrovertible fact, that, although many excellent observations have been recorded, and investigations instituted upon its specific peculiarities, much additional information has yet to be produced.

*Liverpool; October* 31*st*, 1831.

